# Synthetic Mono-Carbonyl Curcumin Analogues Attenuate Oxidative Stress in Mouse Models

**DOI:** 10.3390/biomedicines10102597

**Published:** 2022-10-17

**Authors:** Haya Hussain, Shujaat Ahmad, Syed Wadood Ali Shah, Abid Ullah, Shafiq Ur Rahman, Manzoor Ahmad, Mazen Almehmadi, Osama Abdulaziz, Mamdouh Allahyani, Ahad Amer Alsaiari, Mustafa Halawi, Edrous Alamer

**Affiliations:** 1 Department of Pharmacy, Shaheed Benazir Bhutto University, Sheringal, Dir Upper 18000, Khyber Pakhtunkhwa, Pakistan; 2 Department of Pharmacy, University of Malakand, Chakdara, Dir Lower 18800, Khyber Pakhtunkhwa, Pakistan; 3 Department of Chemistry, University of Malakand, Chakdara, Dir Lower 18800, Khyber Pakhtunkhwa, Pakistan; 4 Department of Clinical Laboratory Sciences, College of Applied Medical Sciences, Taif University, Taif 21944, Saudi Arabia; 5 Department of Medical Laboratory Technology, College of Applied Medical Sciences, Jazan University, Jazan 45142, Saudi Arabia; 6 Medical Research Center, Emerging Infectious Diseases Research Unit, Jazan University, Jazan 45142, Saudi Arabia

**Keywords:** Alzheimer’s disease, oxidative stress, mono-carbonyl curcumin analogues, antioxidants, in vivo study, light–dark box, hole board, Y-maze, biomarkers, hippocampus

## Abstract

Alzheimer’s disease is the commonest form of dementia associated with short-term memory loss and impaired cognition and, worldwide, it is a growing health issue. A number of therapeutic strategies have been studied to design and develop an effective anti-Alzheimer drug. Curcumin has a wide spectrum of biological properties. In this regard, the antioxidant potentials of mono-carbonyl curcumin analogues (**h1**–**h5**) were investigated using in vitro antioxidant assays and hippocampal-based in vivo mouse models such as light–dark box, hole board, and Y-maze tests. In the in vitro assay, mono-carbonyl curcumin analogues **h2** and **h3** with methoxy and chloro-substituents, respectively, showed promising 2,2-diphenyl-1-picrylhydrazyl (DPPH) and 2, 2′-azinobis-3-ethylbenzothiazo-line-6-sulfonate (ABTS) free radical scavenging activities. In the in vivo studies, scopolamine administration significantly (*p* < 0.001) induced oxidative stress and memory impairment in mice, in comparison to the normal control group. The pretreatment with mono-carbonyl curcumin analogues, specifically **h2** and **h3**, significantly decreased (123.71 ± 15.23 s (*p* < 0.001), *n* = 8; 156.53 ± 14.13 s (*p* < 0.001), *n* = 8) the duration of time spent in the light chamber and significantly enhanced (253.95 ± 19.05 s (*p* < 0.001), *n* = 8, and 239.57 ± 9.98 s (*p* < 0.001), *n* = 8) the time spent in the dark compartment in the light–dark box arena. The numbers of hole pokings were significantly (*p* < 0.001, *n* = 8) enhanced in the hole board test and substantially increased the percent spontaneous alternation performance (SAP %) in the Y-maze mouse models in comparison to the stress control group. In the biomarker analysis, the significant reduction in the lipid peroxidation (MDA) level and enhanced catalase (CAT), superoxide dismutase (SOD), and glutathione (GSH) activities in the brain hippocampus reveal their antioxidant and memory enhancing potentials. However, further research is needed to find out the appropriate mechanism of reducing oxidative stress in pathological models.

## 1. Introduction

Alzheimer’s disease (AD) is a neurological disorder linked with many clinical manifestations and a number of biomarkers and environmental and genetic factors are associated with the progression and development of this disease [1,2]. Alzheimer’s disease (AD) is the famous form of dementia, characterized by memory loss, cognitive impairment, and considered a growing health challenge worldwide [3]. Moreover, AD causes death within 3 to 9 years after its diagnosis, and globally, it is reported in about 35 million people [4,5], and will increase to two-fold by 2030, according to a report on AD in 2015 [6]. The exact etiology of the disease is still unknown to researchers. However, the cognitive decline is associated with cholinergic deficiency and it is believed to be the primary cause of AD [5]. Choline acetyl transferase (ChAT) altered the synthesis of acetylcholine (ACh), which was substantially decreased in the brain cerebral cortex of AD, found by three laboratories independently [7] and it was reported in their research that intensity of the cognitive decline increases with a decrease in activity of ChAT in AD patients. This evidence suggests us that cognitive dysfunction is associated with deficiency in the cholinergic system of AD patients [8]. Moreover, deposition of neurofibrillary tau tangles and β-amyloid (Aβ) plaques were also responsible for neurodegeneration [9]. 

Oxidative stress is considered another important contender of the etiological factors for AD associated with oxidative stress by causing pathological interactions with many vital cellular components [10]. Oxidative stress develops as a result of reactive oxygen species (ROS) generation in the form of superoxide, hydroxyl, hydroperoxyl, and alkoxyl as free radicals from metabolic processes [11]. Free radicals have been remained the focus of attention for researchers in the last two decades [12]. These free radicals cause oxidative stress and are associated with serious health conditions such as neurodegenerative diseases, diabetes, cardiovascular diseases, and cancers [13,14]. Oxidative stress develops when the production of oxidants (free radicals) exceeds that of antioxidants and, consequently, it weakens the antioxidant protection systems in the living cells [15], leading to the neuronal degeneration [16]. Oxidative stress is further linked to tau-induced and Abeta neurotoxicity by their overproduction and aggregation; polymerization and phosphorylation processes lead to the progression and development of AD [17,18,19]. Oxidative stress progressively causes neurodegeneration and is responsible for slow tissue regeneration in aged individuals and rodents [20]. In addition, oxidative stress is responsible for the other key characteristics such as cell-cycle, metabolic, and mitochondrial abnormalities found in AD [21]. These conditions lead to the appearance of the clinical symptoms of AD, such as delusions, memory deficits, cognitive dysfunction, depression, apathy, and behavioral disorders [22]. As oxidative stress is associated with many disease conditions, improving the antioxidant system of biological targets is of valuable interest [23]. 

In the central nervous system, scopolamine, a well-known muscarinic receptor blocker of acetylcholine receptors, alters the acetylcholine synthesis and is extensively used in animal models for the induction of amnesia [2,24]. It has been reported that scopolamine administration chronically depletes the antioxidant defense system in cells [25].

There is a complex system of enzymes and antioxidant metabolites working to protect the important cellular components from the damage of reactive oxygen species (ROS) by inhibiting their production or removal from the body [11]. Antioxidants are substances with the capability to protect living cells from oxidative stress by oxidizing themselves, slowing the oxidation reaction, such as ascorbic acid and polyphenols [11,26]. A number of therapeutic strategies and approaches have been adopted to find therapeutic candidates with antioxidant potential as well as to regulate the cholinergic system for the prevention and treatment of AD using animal models [22,27]. In this regard, various cholinesterase inhibitors are in current use, including donepezil, galantamine, tacrine, and revastigmine for relieving symptoms of dementia. However, their efficacy will decrease with long-term use [6,28,29]. In addition, various types of natural and synthetic antioxidants substances work in the management of AD [30]. Natural antioxidants are common in various fruits, vegetables, and herbs [31], while synthetic antioxidants are butylated hydroxyl toluene (BHT), gallic acid ester, butylated hydroxyl anisole (BHA), and butylated hydroquinone [32]. Therefore, the design and development of safe and effective antioxidants are needed through quality research for the effective treatment of neurological disorders, including AD.

Curcumin, from the bright yellow dietary spice turmeric and found in the rhizome of *Curcuma longa* L. [33], possesses a variety of biological properties such as neuroprotective, antioxidant, hypoglycemic, ant-inflammatory, antimicrobial, antiviral, and anticancer, and is also widely used as a dietary pigment and spice [34,35,36,37,38]. Moreover, chronic administration of curcumin gives control over neurochemical and behavioral alterations reported in several preclinical and pharmacological animal models [39].

Based on these observations, our research group have previously reported on these compounds, indicating their neuroprotective potentials by inhibiting cholinesterases [5]. Keeping in mind their neuroprotective profile, this study was designed to find out their antioxidant potential in scopolamine-induced oxidative stress mouse models.

## 2. Materials and Methods

### 2.1. Chemicals and Animals 

**Figure 1:**
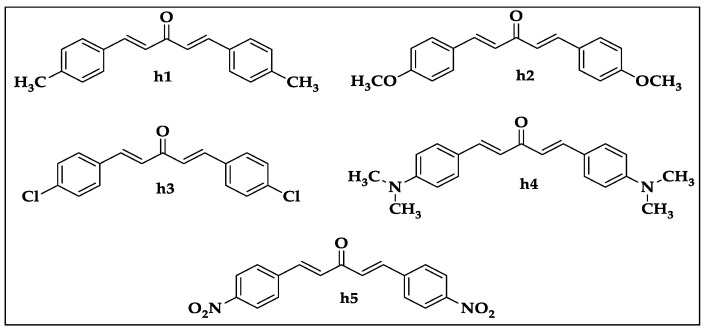
Chemical structures of mono-carbonyl curcumin analogues (**h1**–**h5**).

The chemical structures of the synthesized mono-carbonyl curcumin analogues (**h1**–**h5**) used in this study are illustrated in Figure 1, as previously reported by our research group [5]. 

All the chemicals and reagents used in this research, including DPPH, ABTS, methanol, acetic acid, n-butanol, trichloracetic acid (TCA), reduced nicotinamide adenine dinucleotide (NADH), thiobarbituric acid (TBA), catalase (CAT), superoxide dismutase (SOD), glutathione (GSH), were of analytical grade from Sigma-Aldrich (Merck, Darmstadt, Germany) and purchased from the local market. Scopolamine (butylbromide) and donepezil were obtained from Venous Pharma Pvt. Lahore and Platinum Pharmaceuticals Pvt. Karachi, Pakistan, respectively.

Three-month-old mice (Balb/C) weighing 19–23 gm of both sexes were procured from the “National Institute of Health (NIH), Islamabad”. Mice were housed under a 12 h light and 12 h dark cycle with free access to food and water at 25 ± 3 °C and 55–65% relative humidity in the animal house. All the animals were acclimatized to the laboratory environment for two weeks before running the experimental models. This study was performed as per the approval of the “Departmental Ethical Committee” vide notification (SBBU/IEC-20-02) in accordance with the “Scientific Procedure Issue-I” 2008 animal bylaws of the University of Malakand.

### 2.2. In Vitro Antioxidant Activity

In vitro antioxidant profiles of mono-carbonyl curcumin analogues (**h1**–**h5**) were explored according to the Brand-Williams et al. protocol of 1995 [40], by means of DPPH and ABTS as free radicals. In the assay of DPPH, the tested compounds (**h1**–**h5**) and the standard drug tocopherol were mixed with 31.25–1000 µg/mL concentrations of DPPH solution, and a spectrophotometer at 517 nm was run and the absorbance was recorded. Similarly, the ABTS assay was performed for the tested compounds (**h1**–**h5**) and standard drug in a similar concentration. In this, 0.1 mL of sample and standard solution were mixed with ABTS at the same concentrations and absorbance at 734 nm was noted. Then, IC_50_ values for the tested samples were calculated.

### 2.3. Acute Toxicity Study

The safety of these compounds was ensured by conducting acute toxicity studies in mice to find any possible toxicological properties and select the safe optimum dose of the tested compounds (**h1**–**h5**) for conducting the in vivo studies. The mono-carbonyl curcumin analogues (**h1**–**h5**) were orally administered to different groups of mice in doses of 5, 15, 30, 50, 75, 100, and 150 mg/kg body weight, and all the animals were keenly observed for toxicity indications for 24 h such as convulsions, tremors, motor activity, loss of righting reflex, lacrimation, diarrhea, muscle spasm, salivation, sedation, and hypnosis. Then, animals were further observed for mortality for 72 h. The animals were safe up to a 150 mg/kg body weight dose. Therefore, as per OECD guidelines, a 15 mg/kg dose of the tested samples as 1/10th of 150 mg/kg was chosen as a suitable dose for in vivo studies of the mono-carbonyl curcumin analogues (**h1**–**h5**) [6].

### 2.4. Experimental Design and Animal Dosing

All the animals were divided into eight groups, having eight in each (*n* = 8) and treated as the sample-treated groups, stress control group, standard treatment group, and normal control group for seven days. In this study, the normal control group was given 5 mL/kg (p.o) normal saline; the stress control group was administered 5 mL/kg (p.o) normal saline (2% Tween 80); the standard control group received donepezil 2 mg/kg (p.o); and the sample-treated groups received mono-carbonyl curcumin analogues (**h1**–**h5**) at 15 mg/kg body weight (p.o) suspended in 2% Tween 80, for seven days. On day seven, 30 min after the last dose, scopolamine 1 mg/kg (i.p.) was administered to all groups except the normal control group. Then, the in vivo behavioral activities were performed accordingly.

### 2.5. Behavioral Studies

Oxidative stress was induced in mice using scopolamine in the behavioral mouse models, including light–dark box, hole board, and Y-maze mouse models.

#### 2.5.1. Light–Dark Box

The paradigm of the light–dark box was used for the investigation of behavioral and learning tasks using the mono-carbonyl curcumin analogues (**h1**–**h5**) according to the standard procedure [41]. The test was based on the principle that mice favor the dark compartment. This paradigm consists of two compartments and is made of polyacrylic sheets, with a larger transparent chamber with dimensions of 30 cm × 30 cm × 35 cm attached in parallel position with a smaller dark chamber colored with black and having dimensions of 20 cm × 30 cm × 35 cm. A small opening of 5 cm × 5 cm was made for entrance in the middle of the separating wall of both chambers. Lines, 1 mm apart, were drawn on the floor of both chambers. The mouse was observed for 5 min after being put in the light compartment and the time spent in each chamber was noted. The doses were administered to each mouse accordingly, and after the completion of the last dose, the time spent in each compartment was recorded for two days [42].

#### 2.5.2. Hole Board Test

The hole board paradigm was used for the investigation of the learning behavior with the mono-carbonyl curcumin analogues (**h1**–**h5**) according to the standard procedure [43]. The paradigm of the hole board was designed with a rectangular shape from polyacrylic sheets with dimensions of 35 cm × 45 cm × 45 cm. There were sixteen holes (2 cm in diameter) containing black colored sheets 5 cm above the bottom, which were connected to the internal corners of the box. The numbers of hole pokings were recorded for 5 min after putting the mouse in the middle of the apparatus. After completion of the last dose, animals were observed for two consecutive days [44].

#### 2.5.3. Y-Maze Test

The mono-carbonyl curcumin analogues (**h1**–**h5**) were investigated for attenuation of memory impairment with the help of the Y-maze behavioral mouse model according to the standard protocol [5]. In this test, oxidative stress was induced by injecting 1 mg/kg (i.p.) scopolamine. The paradigm of the Y-maze mice model consists of three equal arms linked with each other at an angle of 120° and designed in a Y-shape, label as A, B, and C for convenience. The arms were 20 cm long, 6 cm wide, and 15.5 cm high. Mice were placed at the center of one arm and allowed to freely explore the apparatus for a period of 5 min. The number of arm entries executed by each mouse was recorded. A complete arm entry is achieved when the hind paws of the animal are totally inside in one of the arms while alternation involved entries made consecutively around the three arms. A solution of ethanol 70% was applied to the Y-maze arena to make it clean and to avoid olfactory cues after every run. The escape latency (seconds) at the end of day seven was noted for each animal. The spontaneous alternation performance (SAP%) was calculated by recording same-arm returns (SARs), alternate arm returns (AARs), and the number of arm entries [6].

### 2.6. Assessment of Biochemical Parameters and Biomarker Study

The biomarker approach was used to find out the antioxidant potential of the mono-carbonyl curcumin analogues (**h1**–**h5**) in oxidative stress, induced by scopolamine with alteration in the endogenous antioxidant enzyme system. Soon after the behavioral analysis, mice were sacrificed by cervical dislocation for a painless death, and the brain tissues were isolated and hippocampus was obtained after homogenization. The hippocampus part of brain tissue was chilled before biomarker analysis in phosphate buffer solution [45].

#### 2.6.1. Measurement of Malondialdehyde (MDA) Level

The malondialdehyde level in the hippocampus was investigated according to the standard procedure [46]. In this method, 100 µL tissue homogenate was added to the mixture of 1.5 mL of thiobarbituric acid (TBA) 0.8%, 1.5 mL of (20%) acetic acid, and 200 µL (8%) sodium dodecyl sulfates. It was then heated for an hour at 90 °C, cooled to room temperature, and added to 5 mL of n-butanol. The mixture was centrifuged for 10 min at 976× *g* and the organic layer was separated out. The MDA level was recorded by measuring absorbance at 532 nm [44].

#### 2.6.2. Catalase (CAT) Activity

The catalase activity was measured in the hippocampus according to the reported procedure of Sinha in 1972 with little modification [47]. Tissue homogenate 0.1 mL was mixed with 1 mL (0.01 M) phosphate buffer pH 7, 5% potassium dichromate–acetic acid 1:3, H_2_O_2_ (2 M), and 2 mL of dichromate acetic acid. The absorbance was measured at 620 nm and the catalase activity was expressed in the µM of H_2_O_2_ decomposing/min/mg of tissue protein [44].

#### 2.6.3. Superoxide Dismutase (SOD) Activity

The superoxide dismutase activity of the mono-carbonyl curcumin analogues was measured according to the standard procedure by Kakkar et al., in 1984 [48], in the mouse hippocampus. The brain tissue homogenate (0.5 mL) dilution was made in 1 mL of distilled water and then shaken with 2.5 mL of ethanol and 1.5 mL of chloroform. The mixture was centrifuged at 4 °C for a period of 1 min. The supernatant was mixed with 1.2 mL (0.025 M) sodium pyrophosphate buffer of 8.4 pH, 0.3 mL (30 µM) NBT, 3 mL distilled water, 0.1 mL (186 µM) PMS, and 0.2 mL (780 µM) NADH. It was then incubated at 30 °C for 90 s and, finally, 1 mL acetic acid was added to stop the reaction. The mixture was stirred vigorously, n-butanol was added, and it was stirred again. Then, the butanol layer was separated out. The absorbance of butanol was measured at 560 nm. The measured SOD amount was presented as a unit/mg of protein [44].

#### 2.6.4. Measurement of Glutathione (GSH) Activity

The effect of mono-curcumin analogues (**h1**–**h5**) on glutathione activity was investigated in the mouse hippocampus according to the reported procedure by Moron et al. in 1979 [49]. First, 0.4 mL (20%) TCA was added to 0.4 mL of the homogenate and centrifuged for 20 min at 10,000× *g* at 4 °C. The supernatant (0.25 mL) was added to 0.6 M (2 mL) DTNB and phosphate buffer pH 8.0 (0.2 M), and the final volume was 3 mL. The GSH activity was recorded at 412 nm absorbance and expressed as µM/mg of protein [44].

### 2.7. Statistical Analysis

Results are presented as mean ± SD, a Kolmogorov–Smirnov test was applied for normality distribution of each data set and one-way ANOVA was used followed by Bonferroni’s multiple comparison tests (post hoc) using statistical package SPSS, version 25. The value of *p* = 0.05 was designated as significant.

## 3. Results

### 3.1. In Vitro Antioxidant Activity

Results of the in vitro antioxidant assay of mono-carbonyl curcumin analogues (**h1**–**h5**) are shown in Table 1. In this assay, the mono-carbonyl curcumin analogues **h2** and **h3** with IC_50_ values of 53.29 ± 2.13 and 82.43 ± 2.17 µg/mL, respectively, showed maximum response against DPPH free radicals in comparison to the positive control with an IC_50_ value of 9.16 ± 1.14 µg/mL. Among other curcumin analogues, **h1** showed moderate free radical scavenging activity with IC_50_ 160.32 ± 1.37 µg/mL, while, **h4** and **h5** showed weaker antioxidant response against DPPH free radicals. Similarly, in the ABTS assay, **h2** with an IC_50_ value of 70.21 ± 1.26 µg/mL showed significant antioxidant response in comparison to the standard drug tocopherol, having an IC_50_ of 13.18 ± 1.16 µg/mL, and **h3** exhibited IC_50_ of 143.65 ± 2.43 µg/mL, showing weaker antioxidant activity, while **h1**, **h4**, and **h5** showed a poor response.

### 3.2. Acute Toxicity

The mono-carbonyl curcumin analogues (**h1**–**h5**) were investigated for possible toxicological effects, and it was observed that up to a maximum administered dose of 150 mg/kg body weight, there was no toxicological response or mortality in mice. After acute toxicity screening, 15 mg/kg b.w. was selected as a suitable dose for the in vivo studies.

### 3.3. In Vivo Behavioral Studies

#### 3.3.1. Light–Dark Box Apparatus

**Figure 2:**
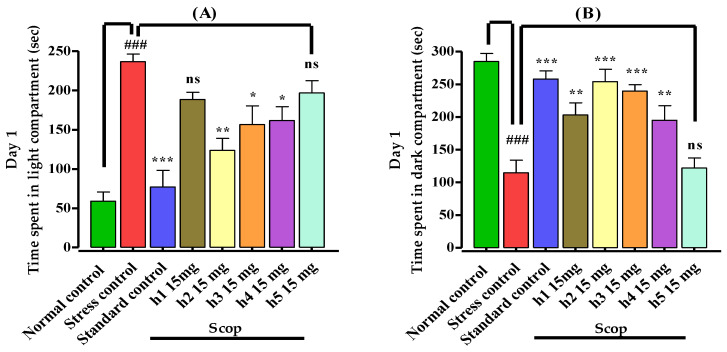

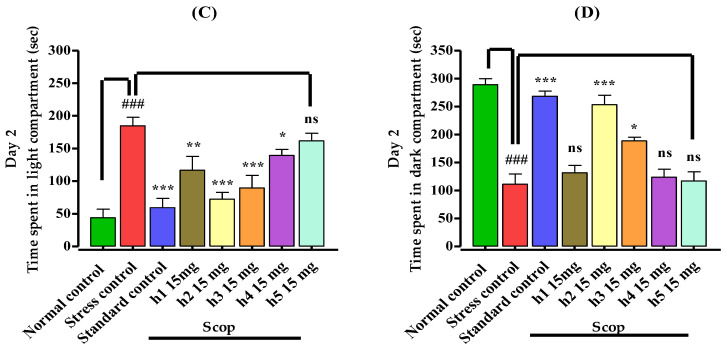
Effect of mono-carbonyl curcumin analogues (**h1**–**h5**) in the light–dark test on (**A**) time spent (s) on day 1 in light compartment, (**B**) time spent (s) on day 1 in dark compartment, (**C**) day 2, time spent (s) in light compartment, and (**D**) day 2, time spent (s) in dark compartment. Results are shown as mean ± SD, *n* = 8. Kolmogorov–Smirnov test was applied for normality distribution and each data set was found to have a normal distribution. One-way ANOVA, followed by Bonferroni’s multiple comparison tests (post hoc), was applied and expressed as ^###^ *p* < 0.001 vs. normal control group, and *** *p* < 0.001, ** *p* < 0.01, * *p* < 0.05, “ns” *p* ˃ 0.05 vs. stress control group.

The effects of mono-carbonyl curcumin analogues (**h1**–**h5**) on the mice’s memory were investigated by using a light–dark box apparatus and the results are shown in Figure 2. Mice in the normal control group stayed for less time in the light chamber and stayed for a longer period in the dark compartment on day 1. Animals in the stress control (amnesic) group remained for a significantly (*p* < 0.001) longer time in the light compartment and spent less time in the dark compartment in comparison to the normal control group, indicating induction of stress. Mice in the treatment groups, treated specifically with the standard drug, and compounds **h2**, **h3**, and **h4**, spent significantly (*p* < 0.001) more time in the dark compartment in comparison to the stress control group, which showed memory improvement by reducing stress (Figure 2A,B). Those treated with compound **h1** spent comparatively (*p* < 0.01) more time in the dark arena and showed memory enhancement effects as compared to the stress control group, while **h5** showed a poor (*p* < 0.05) response on day 1. Similarly, on day 2, those treated with compounds **h2** (*p* < 0.001), **h3** (*p* < 0.001), **h1** (*p* < 0.01), **h4**, and **h5** (*p* ˃ 0.05) remained for a short period in the light chamber and those treated with compounds **h2** (*p* < 0.001) and **h3** (*p* < 0.01) spent significantly more time in the dark chamber, revealing a memory enhancement effect as compared to the stress control group, while **h1**, **h4**, **h5** (*p* ˃ 0.05) showed a poor response (Figure 2C,D).

#### 3.3.2. Hole Board Assay

**Figure 3:**
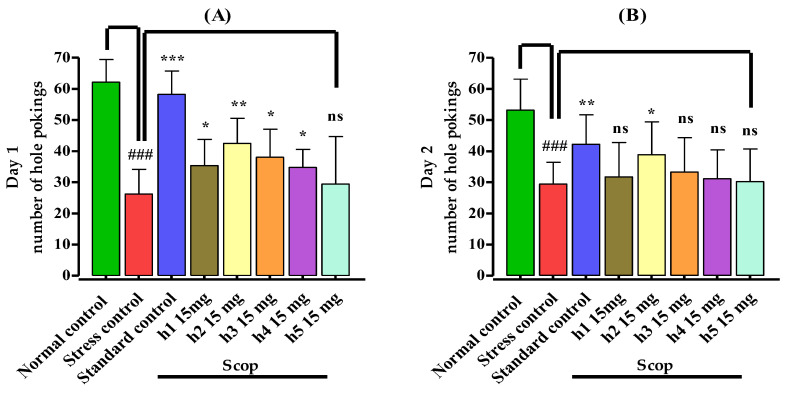
Effect of mono-carbonyl curcumin analogues (**h1**–**h5**) on (**A**) no. of hole pokings on day 1 and (**B**) no. of hole pokings on day 2. Results are shown as mean ± SD, *n* = 8. Kolmogorov–Smirnov test was applied for normality distribution and each data set was found to have a normal distribution. One-way ANOVA, followed by Bonferroni’s multiple comparison tests (post hoc), was applied on each data set and expressed as ^###^ *p* < 0.001 vs. normal control group, and *** *p* < 0.001, ** *p* < 0.01, * *p* < 0.05, “ns” *p* ˃ 0.05 vs. stress control group.

The numbers of hole pokings were significantly (*p* < 0.001) decreased by the administration of scopolamine in comparison to the normal control group, which showed oxidative stress (amnesia) in the stress control group on days 1 and 2, as shown in Figure 3A,B. The mice in the standard control group showed a significantly increased number of hole pokings (*p* < 0.001) on day 1 and (*p* < 0.01) on day 2, showing a reduction in amnesia. Similarly, compound **h2** significantly (*p* < 0.01) increased the number of hole pokings, followed by **h1**, **h3**, **h4** (*p* < 0.05), indicating its antiamnesic potentials, while **h5** (*p* ˃ 0.05) showed no prominent response on day 1. On day 2, only compound **h2** (*p* < 0.05) comparatively increased the number of hole pokings while the rest of the compounds showed no prominent effects on hole pokings in comparison to the stress control group.

#### 3.3.3. Y-Maze Test

**Figure 4:**
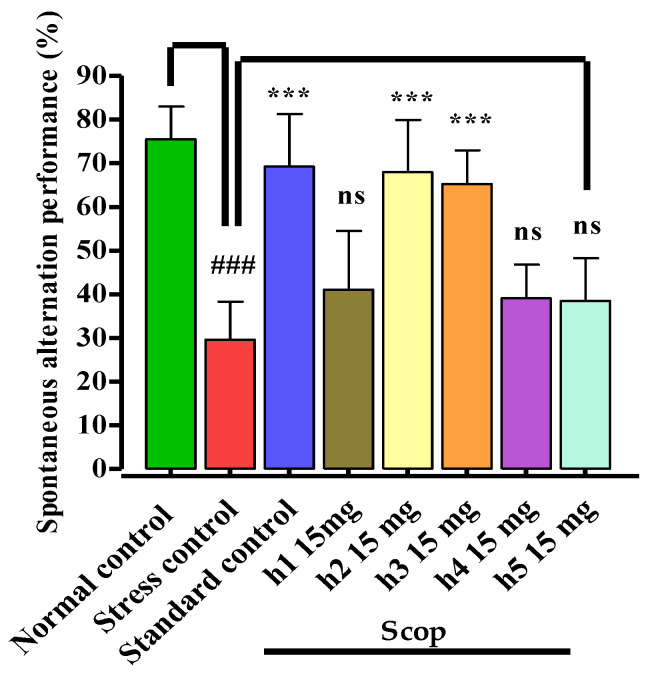
Effects of mono-carbonyl curcumin analogues (**h1**–**h5**) on percent spontaneous alternation performance in Y-maze behavioral mouse model. Results are shown as mean ± SD, *n* = 8. Kolmogorov–Smirnov test was applied for normality distribution and each data set was found to have a normal distribution. One-way ANOVA, followed by Bonferroni’s multiple comparison tests (post hoc), was applied on each data set and expressed as ^###^ *p* < 0.001 vs. normal control group, and *** *p* < 0.001, “ns” *p* ˃ 0.05 vs. stress control group.

The mono-carbonyl curcumin analogues (**h1**–**h5**) were investigated for memory-enhancing effects induced by scopolamine in the Y-maze mouse model (Figure 4). In the stress control group, scopolamine 1 mg/kg (i.p.) administration caused significant (*p* < 0.001) memory deficits in the stress control group. The standard control and compounds **h2** (*p* < 0.001) and **h3** (*p* < 0.001) significantly reversed the scopolamine-induced memory deficits by enhancing the percent spontaneous alternation performance in mice. Similarly, compounds **h1**, **h4**, and **h5** (*p* ˃ 0.05) showed poor response in comparison to the stress control group. The results indicated that mono-carbonyl curcumin analogues, specifically **h2** and **h3**, showed memory-enhancing effects by reversing the memory deficits caused by scopolamine administration.

### 3.4. Assessment of Biochemical Parameters and Biomarker Study

**Figure 5:**
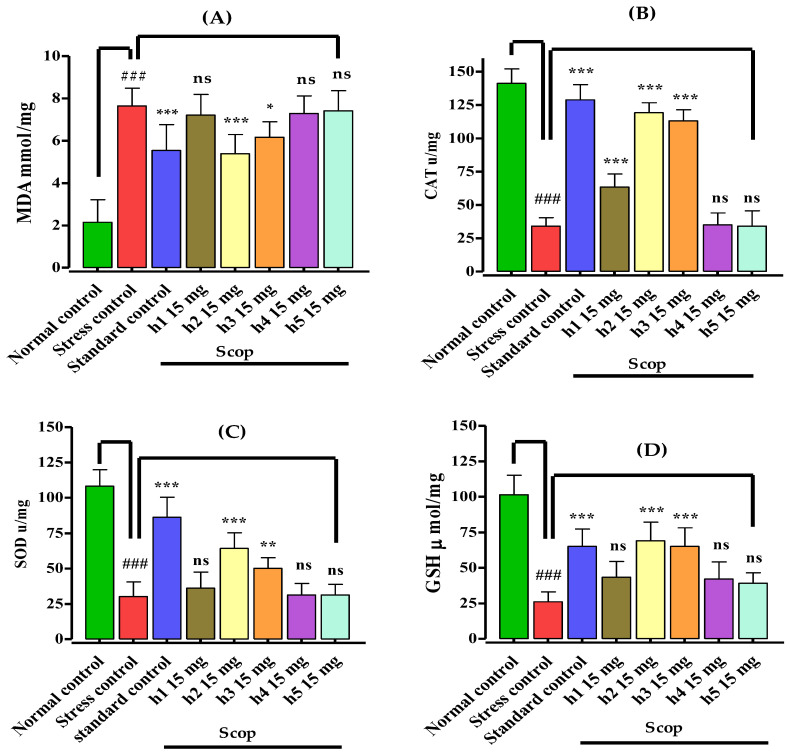
Effect of mono-carbonyl curcumin analogues (**h1**–**h5**) on the (**A**) malondialdehyde (MDA) level, (**B**) catalase (CAT) activity, (**C**) superoxide dismutase (SOD) activity, (**D**) glutathione (GSH) activity in the hippocampus while donepezil served as standard control. Results are shown as mean ± SD, *n* = 8. Kolmogorov–Smirnov test was applied for normality distribution and each data set was found to have a normal distribution. One-way ANOVA, followed by Bonferroni’s multiple comparison tests (post hoc), was applied on each data set and expressed as ^###^ *p* < 0.001 vs. normal control group, and *** *p* < 0.001, ** *p* < 0.01, * *p* < 0.05, “ns” *p* ˃ 0.05 vs. stress control group.

The mono-carbonyl curcumin analogues (**h1**–**h5**) after the in vivo behavioral studies were immediately subjected to biochemical assessment and biomarker studies. The isolated, homogenized hippocampus was analyzed for the determination of the activity of antioxidant enzymes and the level of malondialdehyde. In this study, scopolamine administration caused a reduction in the antioxidant enzymes and increased the MDA level, which indicated the induction of oxidative stress (Figure 5).

The administration of scopolamine 1 mg/kg (i.p.) induced oxidative stress and caused a significant (*p* < 0.001) increase in the lipid peroxidation (MDA) level in comparison to the normal control group (Figure 5A). Pretreatment with mono-carbonyl curcumin analogues for seven days reversed the oxidative stress by reducing the MDA level. The standard drug donepezil and **h2** significantly (*p* < 0.001) decreased the MDA level in comparison to the stress control group. Curcumin analogue **h3** moderately (*p* < 0.05) reduced the MDA level as compared to the stress control group. Likewise, in this assay, compounds **h1**, **h4**, and **h5** showed no promising response.

In the catalase (CAT) assay, injecting scopolamine 1 mg/kg (i.p.) induced oxidative stress in the mice of the stress control group by significantly (*p* < 0.001) decreasing the activity of catalase in the hippocampus in comparison to the normal control group (Figure 5B). Pretreatment with compounds **h1**, **h2**, **h3** and standard control significantly (*p* < 0.001) increased the catalase activity in the hippocampus in comparison to the stress control group, indicating their antioxidant potential. Compounds **h4** and **h5** did not show a significant (*p* ˃ 0.05) increase in catalase level and showed comparative antioxidant response as compared to the stress control group, while **h4** and **h5** showed no promising (*p* *˃* 0.05) antioxidant activity.

The study of superoxide dismutase (SOD) in the hippocampus of the mouse brain indicated that upon administration of scopolamine 1 mg/kg (i.p.), oxidative stress was induced in the hippocampus in the animals of the stress control group (Figure 5C). This revealed the significant (*p* < 0.001) reduction in SOD activity, which reverted the memory deficits with the pretreatment of consecutive doses for seven days of the standard drug (*p* < 0.001), **h2** (*p* < 0.001), and h3 (*p* < 0.01), in comparison to the stress control group, indicating that these types of curcumin analogues with methoxy and chloro substituent have strong antioxidant potential, while compound **h1** with methyl, **h4** with N,N dimethyl substituent, and **h5** with nitro substituent showed no promising (*p* *˃* 0.05) antioxidant activity in comparison to the stress control group.

Similarly, in the assay of glutathione (GSH), the activity of GSH was significantly (*p* < 0.001) lower upon the administration of scopolamine in the hippocampus of mice in the stress control group in comparison to the normal control group, indicating induction of oxidative stress (Figure 5D). The standard (*p* < 0.001) and the mono-carbonyl curcumin analogues **h2** (*p* < 0.001) and **h3** (*p* < 0.001) significantly increased the GSH activity in the hippocampus in comparison to the stress control group, which indicated the reversal of oxidative stress and showed the antioxidant potential of these compounds in mouse models, while compounds **h1**, **h4**, and **h5** (*p* ˃ 0.05) showed no promising antioxidant responses.

## 4. Discussion

The reactive oxygen species caused oxidative stress which is responsible for neurodegenerative diseases [50], and it has been reported that oxidative stress plays a pivotal role as one of the etiologic factors of dementia and more than a hundred other diseases [44,51]. The main causes of memory loss in Alzheimer’s disease (AD) are severe oxidative damage and alterations in the cholinergic system in the brain, deposition of β-amyloid protein, and extra neuronal plaque development [44]. Scopolamine, a muscarinic ACh receptor blocker, is extensively used in rodents and humans to induce oxidative stress, alter the neuronal cholinergic pathway, and reduce hippocampal volume [22]. Scopolamine quickly crosses the blood–brain barrier, causing induction of antimuscarinic activity by depleting acetylcholine, leading to memory loss. Thus, scopolamine is widely used for the induction of dementia in experimental animal models to investigate new compounds for assessing memory and learning behaviors [24].

Those agents which prevent oxidative stress and increase cholinergic activity in the neurons of the brain could be used to reduce the severity of dementia [52]. The symptomatic relief in dementia is provided by activating the cholinergic system by using cholinesterase blockers. At present, galantamine, donepezil, tacrine, and rivastigmine are the approved cholinesterase inhibitors recently reported to provide symptomatic relief in AD [6,28,29]. These only manage the severity of the illness and do not cure the disease completely due to their diminished long-term efficacy and are often associated with side effects. Therefore, the design and development of an effective agent for the treatment of AD are urgently required [6]. In this scenario, our initial study reported on mono-carbonyl curcumin analogues (**h1**–**h5**) proven to be effective acetylcholine esterase inhibitors in animal models [5]. These compounds showed significant antioxidant potential in the in vitro assay. Research studies indicated that methoxy (–OCH_3_) and hydroxyl (-OH) substituted derivatives are responsible for the their antioxidant effects [53]. The mono-carbonyl curcumin analogues with a methoxy substituted moiety exhibited significant antioxidant potential and this was consistent with reported studies [54,55]**.**

The light–dark box mouse model works on the principle of the animal behavior of avoiding the light chamber and exploring the novel environment [56]. The memory assessment using the Y-maze test works on the principle of rodents exploring objects and is considered one of the most convenient and reliable behavior models [57]. The Y-maze mouse model records the percent of spontaneous alternation behavior in mice [6]. Similarly, the head dips and rearing (hole poking) behaviors in mice in the hole board test were used for the assessment of anxiety-like behavior [58,59]. The time spent in the light chamber was longer upon administration of scopolamine, indicating the induction of oxidative stress. The reduced tendency of animals to enter the light compartment, the decrease in the number of hole pokings, and the reduction in the percent spontaneous alternation performance represent a condition of fear, anxiety, and stress in the light–dark box, hole board, and Y-maze mouse models, respectively [5,41,60]. The mono-carbonyl curcumin analogues in the in vivo models significantly decreased the time spent in the light chamber and increased the time spent in the dark compartment in the light–dark box test. The numbers of holes pokings were higher in the hole board test and the percent spontaneous alternation performance was higher in the Y-maze test. The reported curcumin analogues showed promising results and increased the percent spontaneous alternation performance. These results were consistent with previous findings [6,61].

The administration of scopolamine was responsible for the brain’s reduced antioxidant capacity and a marked increase in the MDA level, which is considered the index of lipid peroxidation. Scopolamine caused an increase in the markers of oxidative stress and memory impairment by reducing the cholinergic neurotransmission in the brain [62]. It has been reported that during stressful situations the nervous system is often prone to enhanced lipid peroxidation levels due to high oxygen tension and oxidative damage [45]. In addition, injecting scopolamine in rodents weakens the antioxidant system and induces the lipid peroxidation in the brain, including in those brain parts responsible for memory and learning phenomena [63]. In conditions of severe oxidative stress, the increased generation of reactive oxygen species interacts with many vital components of cells, including lipids, carbohydrates, nucleic acids, and proteins, causing damage to neuronal cells, leading to memory impairment [24]. Similarly, our research showed that the standard drug, donepezil, significantly attenuated the oxidative stress by protecting the antioxidant system and this was consistent with reported studies [64]. These outcomes also suggest the significant antioxidant potential of donepezil, which is important for the attenuation of oxidative stress related to neurodegenerative disorders [65].

In this study, oxidative stress was induced by injecting scopolamine, appearing in the form of disorderly markers of oxidative stress such as higher lipid peroxidation (MDA) and a reduction in the antioxidant enzyme system catalase (CAT), superoxide dismutase (SOD), and glutathione (GSH), responsible for memory loss [62]. The more oxygen is consumed, the more the nervous system is prone to enhanced MDA levels during stressful circumstances [45,66]. The reduction in lipid peroxidation with pretreatment of mono-carbonyl curcumin analogues indicated their antioxidant potential by reducing oxidative stress in mice. It has been reported in various studies that administration of curcumin causes a reduction in oxidative stress [38,67]. The scopolamine administration not only increased the lipid peroxidation but also reduced the activity of antioxidant systems in the brain such as CAT, SOD, and GSH [22,68]. The reported mono-carbonyl curcumin analogues substantially altered the effect of scopolamine and increased the activity of CAT, SOD, and GSH by reducing the oxidative stress in the hippocampus, and these findings were consistent with a reported study [38]. At present, treating Alzheimer’s disease and other neurological disorders with curcumin is a focus of interest for researchers [37]. Chronic administration of curcumin analogues improves neurogenesis [69]. Bearing in mind the concluding results of this work, mono-carbonyl curcumin analogues reduce the severity of dementia as well as improve cognition.

Despite its tremendous pharmacological profile, curcumin has poor water solubility, low permeability through the BBB, and poor bioavailability, which will make it difficult for it to be an ideal drug [70]. Our reported curcumin analogues have also been found to be poorly water soluble. The low bioavailability of curcumin is considered one of the main obstacles for its clinical application against various disorders such as AD and cancers [71]. There are various strategies, such as nano-formulations [72] and the synthesis of curcumin analogues with structural modification and also combination with other multiple components, to enhance its efficacy. These strategies will not only improve the efficacy of curcumin and its analogues but will also enhance its therapeutic effectiveness against a number of diseases such as AD.

## 5. Limitations

The current research work involved in vitro and hippocampal-based investigation. Further research studies are suggested to explore the complete pharmacological profiles, specifically neuropharmacological, of these compounds to find a molecule that can be added to the therapy of oxidative stress-induced neurodegenerative diseases such as Alzheimer’s disease (AD).

## 6. Conclusions

In conclusion, oxidative stress is responsible for altering the endogenous antioxidant markers and is associated with reduced cholinergic system activity, leading to dementia. The mono-carbonyl curcumin analogues decrease oxidative stress and show their effectiveness in attenuating memory loss associated with dementia in mice. This study suggested that the neuroprotective effect of these compounds on scopolamine-induced oxidative stress was due to improving the antioxidant and cholinergic systems in the hippocampus. In addition, these compounds might be appropriate candidates for treating stress-induced cognitive dysfunction after exploring their complete pharmacological profile.

## Figures and Tables

**Table 1:** In vitro antioxidant activity of mono-carbonyl curcumin analogues (**h1**–**h5**).

Compound	DPPH (IC_50_ µg/mL)	ABTS (IC_50_ µg/mL)
h1	160.32 ± 1.37	184.19 ± 1.37
h2	53.29 ± 2.13	70.21 ± 1.26
h3	82.43 ± 2.17	143.65 ± 2.43
h4	230.18 ± 2.15	356.38 ± 1.17
h5	357.13 ± 1.16	435.31 ± 1.18
Tocopherol	9.16 ± 1.14	13.18 ± 1.16

Data are presented as mean ± SEM (*n* = 3), the significantly different values were compared to the positive control.

## Data Availability

Data is contained within the article.

## References

[1] Thomas D.X., Bajaj S., McRae-McKee K., Hadjichrysanthou C., Anderson R.M., Collinge J. (2020). Association of TDP-43 proteinopathy, cerebral amyloid angiopathy, and Lewy bodies with cognitive impairment in individuals with or without Alzheimer’s disease neuropathology. Sci. Rep..

[2] Capatina L., Todirascu-Ciornea E., Napoli E.M., Ruberto G., Hritcu L., Dumitru G. (2020). Thymus vulgaris essential oil protects zebrafish against cognitive dysfunction by regulating cholinergic and antioxidants systems. Antioxidants.

[3] Stanciu G.D., Luca A., Rusu R.N., Bild V., Ioan S., Chiriac B., Solcan C., Bild W., Ababei D.C. (2020). Alzheimer’s Disease Pharmacotherapy in Relation to Cholinergic System Involvement. Biomolecules.

[4] Shal B., Khan A., Khan A.U., Ullah R., Ali G., Islam S.U., Haq I.U., Ali H., Seo E.K., Khan S. (2021). Alleviation of memory deficit by bergenin via the regulation of reelin and Nrf-2/NF-κB pathway in transgenic mouse model. Int. J. Mol. Sci..

[5] Hussain H., Ahmad S., Wadood S., Shah A., Ghias M., Ullah A., Rahman S.U., Kamal Z., Khan F.A., Khan N.M. (2021). Neuroprotective Potential of Synthetic Mono-Carbonyl Curcumin Analogs Assessed by Molecular Docking Studies. Molecules.

[6] Hussain H., Ahmad S., Wadood S., Shah A., Ullah A., Ali N., Almehmadi M., Ahmad M., Ali A., Khalil K. (2022). Attenuation of Scopolamine-Induced Amnesia via Cholinergic Modulation in Mice by Synthetic Curcumin Analogs. Molecules.

[7] Bergmann K., Tomlinson B.E., Blessed G., Gibson P.H., Perry R.H. (1978). Correlation of cholinergic abnormalities with senile plaques and mental test scores in senile dementia. Br. Med. J..

[8] Sugimoto H. (2008). The new approach in development of anti-Alzheimer’s disease drugs via the cholinergic hypothesis. Chem. Biol. Interact..

[9] Hammond T.C., Xing X., Wang C., Ma D., Nho K., Crane P.K., Elahi F., Ziegler D.A., Liang G., Cheng Q. (2020). β-amyloid and tau drive early Alzheimer’s disease decline while glucose hypometabolism drives late decline. Commun. Biol..

[10] Alisi I.O., Uzairu A., Abechi S.E., Idris S.O. (2018). Quantitative structure activity relationship analysis of coumarins as free radical scavengers by genetic function algorithm. Phys. Chem. Res..

[11] Shoaib M., Shah S.W.A., Ali N., Shah I., Naveed Umar M., Shafiullah, Ayaz M., Tahir M.N., Akhtar S. (2015). In vitro enzyme inhibition potentials and antioxidant activity of synthetic flavone derivatives. J. Chem..

[12] Valko M., Rhodes C.J., Moncol J., Izakovic M., Mazur M. (2006). Free radicals, metals and antioxidants in oxidative stress-induced cancer. Chem. Biol. Interact..

[13] Ighodaro O.M., Akinloye O.A. (2018). First line defence antioxidants-superoxide dismutase (SOD), catalase (CAT) and glutathione peroxidase (GPX): Their fundamental role in the entire antioxidant defence grid. Alexandria J. Med..

[14] Spiegel M., Kapusta K., Kołodziejczyk W., Saloni J., Zbikowska B., Hill G.A., Sroka Z. (2020). Antioxidant Activity of Selected Phenolic Acids–Ferric Reducing Antioxidant Power Assay and QSAR Analysis of the Structural Features. Molecules.

[15] Kancheva V.D., Dettori M.A., Fabbri D., Alov P., Angelova S.E., Slavova-Kazakova A.K., Carta P., Menshov V.A., Yablonskaya O.I., Trofimov A.V. (2021). Natural chain-breaking antioxidants and their synthetic analogs as modulators of oxidative stress. Antioxidants.

[16] Peng Y., Chang X., Lang M. (2021). Iron homeostasis disorder and alzheimer’s disease. Int. J. Mol. Sci..

[17] Persson T., Popescu B.O., Cedazo-Minguez A. (2014). Oxidative stress in Alzheimer’s disease: Why did antioxidant therapy fail?. Oxid. Med. Cell. Longev..

[18] Zhao Y., Zhao B. (2013). Oxidative Stress and the Pathogenesis of Alzheimer’s Disease. Oxid. Med. Cell. Longev..

[19] Huang W.J., Zhang X., Chen W.W. (2016). Role of oxidative stress in Alzheimer’s disease (review). Biomed. Rep..

[20] Vanzella C., Neves J.D., Vizuete A.F., Aristimunha D., Kolling J., Longoni A., Gonçalves C.A.S., Wyse A.T.S., Netto C.A. (2017). Treadmill running prevents age-related memory deficit and alters neurotrophic factors and oxidative damage in the hippocampus of Wistar rats. Behav. Brain Res..

[21] Nunomura A., Castellani R.J., Zhu X., Moreira P.I., Perry G., Smith M.A. (2006). Involvement of oxidative stress in Alzheimer disease. J. Neuropathol. Exp. Neurol..

[22] Pathway B.T., Baek S.Y., Li F.Y., Kim D.H., Kim S.J., Kim M.R. (2020). Enteromorpha prolifera Extract Improves Memory in Scopolamine-Treated Mice via Downregulating Amyloid-β Expression and Upregulating. Antioxidants.

[23] Slavova-Kazakova A., Angelova S., Fabbri D., Antonietta Dettori M., Kancheva V.D., Delogu G. (2021). Antioxidant properties of novel curcumin analogues: A combined experimental and computational study. J. Food Biochem..

[24] Rabiei Z., Setorki M. (2018). Effect of hydroalcoholic echium amoenum extract on scopolamine-induced learning and memory impairment in rats. Pharm. Biol..

[25] Fronza M.G., Baldinotti R., Fetter J., Sacramento M., Sabedra F.S., Seixas F.K., Collares T., Alves D., Pratico D., Savegnago L. (2020). QTC-4-MeOBnE rescues scopolamine-induced memory deficits in mice by targeting oxidative stress, neuronal plasticity and apoptosis QTC-4-MeOBnE rescues scopolamine-induced memory deficits in mice by targeting oxidative stress, neuronal plasticity and apo. ACS Chem. Neurosci..

[26] Mošovská S., Petáková P. (2016). Antioxidant properties of curcuminoids isolated from *Curcuma longa* L. Acta Chim. Slovaca.

[27] Lee J.E., Song H.S., Park M.N., Kim S.H., Shim B.S., Kim B., Lee J.E. (2018). Ethanol Extract of Oldenlandia diffusa Herba Attenuates Scopolamine-Induced Cognitive Impairments in Mice via Activation of BDNF, P-CREB and Inhibition of Acetylcholinesterase. Int. J. Mol. Sci..

[28] Castro A., Martinez A. (2006). Targeting Beta-Amyloid Pathogenesis Through Acetylcholinesterase Inhibitors. Curr. Pharm. Des..

[29] Orhan G., Orhan I., Sener B. (2006). Recent Developments in Natural and Synthetic Drug Research for Alzheimers Disease. Lett. Drug Des. Discov..

[30] Sadiq A., Mahmood F., Ullah F., Ayaz M., Ahmad S., Haq F.U., Khan G., Jan M.S. (2015). Synthesis, anticholinesterase and antioxidant potentials of ketoesters derivatives of succinimides: A possible role in the management of alzheimer’s. Chem. Cent. J..

[31] Zheng W., Wang S.Y. (2001). Antioxidant Activity and Phenolic Compounds in Selected Herbs. J. Agric. Food Chem..

[32] Von Gadow A., Joubert E., Hansmann C.F. (1997). Comparison of the Antioxidant Activity of Aspalathin with That of Other Plant Phenols of Rooibos Tea (Aspalathus linearis), r-Tocopherol, BHT, and BHA. J. Agric. Food Chem..

[33] Mbese Z., Khwaza V., Aderibigbe B.A. (2019). Curcumin and Its Derivatives as Potential Therapeutic Agents in Prostate, Colon and Breast Cancers. Molecules.

[34] Liang G., Yang S., Jiang L., Zhao Y., Shao L., Xiao J., Ye F., Li Y., Li X. (2008). Synthesis and anti-bacterial properties of mono-carbonyl analogues of curcumin. Chem. Pharm. Bull..

[35] Forms P. (2022). Curcumin: Biological Activities and Modern. Antibiotics.

[36] Ahmed T., Gilani A.H. (2009). Inhibitory effect of curcuminoids on acetylcholinesterase activity and attenuation of scopolamine-induced amnesia may explain medicinal use of turmeric in Alzheimer’s disease. Pharmacol. Biochem. Behav..

[37] Lee W., Loo C., Bebawy M., Luk F., Mason R.S. (2013). Curcumin and its Derivatives: Their Application in Neuropharmacology and Neuroscience in the 21 st Century. Curr. Neuropharmacol..

[38] Naqvi F., Haider S., Naqvi F., Saleem S., Perveen T., Batool Z. (2019). A comparative study showing greater effects of curcumin compared to donepezil on memory function in rats. Pak. J. Pharm. Sci..

[39] Zhang Y., Li L., Zhang J. (2020). Curcumin in antidepressant treatments: An overview of potential mechanisms, pre-clinical/clinical trials and ongoing challenges. Basic Clin. Pharmacol. Toxicol..

[40] Brand-Williams W., Cuvelier M.E., Berset C. (1995). Use of a Free Radical Method to Evaluate Antioxidant Activity. Food Sci. Technol..

[41] Barry J.M., Costall B., Kelly M.E., Naylor R.J. (1987). Withdrawal syndrome following subchronic treatment with anxiolytic agents. Pharmacol. Biochem. Behav..

[42] Kaufmann F.N., Gazal M., Bastos C.R., Kaster M.P., Ghisleni G. (2016). Curcumin in depressive disorders: An overview of potential mechanisms, preclinical and clinical findings. Eur. J. Pharmacol..

[43] Durcan M.J., Lister R.G. (1988). Time course of ethanol’s effects on locomotor activity, exploration and anxiety in mice. Psychopharmacology.

[44] Mushtaq A., Anwar R., Ahmad M. (2018). *Lavandula stoechas* (L) a very potent antioxidant attenuates dementia in scopolamine induced memory deficit mice. Front. Pharmacol..

[45] Ghias M., Wadood S., Shah A., Al-joufi F.A., Shoaib M., Muhammad S., Shah M., Ahmed M.N., Zahoor M. (2022). In Vivo Antistress Effects of Synthetic Flavonoids in Mice: Behavioral and Biochemical Approach. Molecules.

[46] Ohkawa H., Ohishi N., Yagi K. (1979). Assay for lipid peroxides in animal tissues by thiobarbituric acid reaction. Anal. Biochem..

[47] Sinha A.K. (1972). Colorimetric assay of catalase. Anal. Biochem..

[48] Kakkar P., Das B., Viswanathan P.N. (1984). A modified spectrophotometric assay of superoxide dismutase. Indian J. Biochem. Biophys..

[49] Moron M.S., Depierre J.W., Mannervik B. (1979). Levels of glutathione, glutathione reductase and glutathione S-transferase activities in rat lung and liver. Biochim. Biophys. Acta.

[50] Rawat N., Ahmad Y., Kant R., Singh T.G., Arora G., Dhiman S., Singh T.G. (2019). Anti-amnesic activity of ascophyllum nodosum polyphenols on trihexyphenidyl induced amnesia. Plant Arch..

[51] Ramos S., Filho S., Haroldo J., Barbosa O., Rondinoni C., Carlos A., Ernesto C., Salmon G., Kilza N., Ferriolli E. (2017). Neuro-degeneration profile of Alzheimer’s patients: A brain morphometry study. NeuroImage Clin. J..

[52] Rajesh V., Riju T., Venkatesh S., Babu G. (2017). Memory enhancing activity of Lawsonia inermis Linn. leaves against scopolamine induced memory impairment in Swiss albino mice. Orient. Pharm. Exp. Med..

[53] Zafar R., Ullah H., Zahoor M., Sadiq A. (2019). Isolation of bioactive compounds from *Bergenia ciliata* (haw.) Sternb rhizome and their antioxidant and anticholinesterase activities. BMC Complement. Altern. Med..

[54] Khushwant S. (2013). Bhullar Curcumin and Its Carbocyclic Analogs: Structure-Activity in Relation to Antioxidant and Selected Biological Properties. Molecules.

[55] Anand P., Thomas S.G., Kunnumakkara A.B., Sundaram C., Harikumar K.B., Sung B., Tharakan S.T., Misra K., Priyadarsini I.K., Rajasekharan K.N. (2008). Biological activities of curcumin and its analogues (Congeners) made by man and Mother Nature. Biochem. Pharmacol..

[56] Bourin M., Hascoët M. (2003). The mouse light/dark box test. Eur. J. Pharmacol..

[57] Ru M., Liu H. (2018). Association between Y-Maze Acquisition Learning and Major Histocompatibility Complex Class II Polymorphisms in Mice. BioMed Res. Int..

[58] Walf A.A., Frye C.A. (2007). The use of the elevated plus maze as an assay of anxiety-related behavior in rodents. Nat. Protoc..

[59] Kumar N., Singh N., Jaggi A.S. (2012). Anti-stress effects of cilnidipine and nimodipine in immobilization subjected mice. Physiol. Behav..

[60] Rubab S., Naeem K., Rana I., Khan N., Afridi M., Ullah I., Shah F.A., Sarwar S., Din F., Choi H.I. (2021). Enhanced neuroprotective and antidepressant activity of curcumin-loaded nanostructured lipid carriers in lipopolysaccharide-induced depression and anxiety rat model. Int. J. Pharm..

[61] Lee G.Y., Lee C., Park G.H., Jang J.H. (2017). Amelioration of Scopolamine-Induced Learning and Memory Impairment by α-Pinene in C57BL/6 Mice. Evid.-Based Complement. Altern. Med..

[62] Budzynska B., Boguszewska-czubara A., Kruk-slomka M., Skalicka-wozniak K., Michalak A., Musik I., Biala G. (2015). Effects of imperatorin on scopolamine-induced cognitive impairment and oxidative stress in mice. Psychopharmacology.

[63] Kwon S.H., Lee H.K., Kim J.A., Hong S.I., Kim H.C., Jo T.H., Park Y.I., Lee C.K., Kim Y.B., Lee S.Y. (2010). Neuroprotective effects of chlorogenic acid on scopolamine-induced amnesia via anti-acetylcholinesterase and anti-oxidative activities in mice. Eur. J. Pharmacol..

[64] Saxena G., Singh S.P., Agrawal R., Nath C. (2008). Effect of donepezil and tacrine on oxidative stress in intracerebral streptozotocin-induced model of dementia in mice. Eur. J. Pharmacol..

[65] Umukoro S., Adewole F.A., Eduviere A.T., Aderibigbe A.O., Onwuchekwa C. (2014). Free radical scavenging effect of donepezil as the possible contribution to its memory enhancing activity in mice. Drug Res..

[66] Nade V.S., Kawale L.A., Naik R.A., Yadav A.V. (2009). Adaptogenic effect of Morus alba on chronic footshock-induced stress in rats. Indian J. Pharmacol..

[67] Hatcher H., Planalp R., Cho J., Torti F.M., Torti S.V. (2008). Curcumin: From ancient medicine to current clinical trials. Cell. Mol. Life Sci..

[68] Du C.N., Min A.Y., Kim H.J. (2015). Deer bone extract prevents against scopolamine-induced memory impairment in mice. J. Med. Food.

[69] First M., Gil-Ad I., Taler M., Tarasenko I., Novak N., Weizman A. (2011). The effects of fluoxetine treatment in a chronic mild stress rat model on depression-related behavior, brain neurotrophins and ERK expression. J. Mol. Neurosci..

[70] He X., Zhu Y., Wang M., Jing G., Zhu R., Wang S. (2016). Antidepressant effects of curcumin and HU-211 coencapsulated solid lipid nanoparticles against corticosterone-induced cellular and animal models of major depression. Int. J. Nanomed..

[71] Teiten M.H., Dicato M., Diederich M. (2014). Hybrid curcumin compounds: A new strategy for cancer treatment. Molecules.

[72] Tabanelli R., Brogi S., Calderone V. (2021). Improving curcumin bioavailability: Current strategies and future perspectives. Pharmaceutics.

